# Neuronal guidance proteins in cardiovascular inflammation

**DOI:** 10.1007/s00395-021-00847-x

**Published:** 2021-01-29

**Authors:** Marius Keller, Valbona Mirakaj, Michael Koeppen, Peter Rosenberger

**Affiliations:** Department of Anesthesiology and Intensive Care Medicine, University Hospital Tübingen, Eberhard-Karls-University, Hoppe-Seyler-Strasse 3, 72076 Tübingen, Germany

**Keywords:** Neuronal guidance proteins, Cardiovascular inflammation, Atherosclerosis, Myocardial infarction

## Abstract

Cardiovascular pathologies are often induced by inflammation. The associated changes in the inflammatory response influence vascular endothelial biology; they complicate the extent of ischaemia and reperfusion injury, direct the migration of immune competent cells and activate platelets. The initiation and progression of inflammation is regulated by the classical paradigm through the system of cytokines and chemokines. Therapeutic approaches have previously used this knowledge to control the extent of cardiovascular changes with varying degrees of success. Neuronal guidance proteins (NGPs) have emerged in recent years and have been shown to be significantly involved in the control of tissue inflammation and the mechanisms of immune cell activation. Therefore, proteins of this class might be used in the future as targets to control the extent of inflammation in the cardiovascular system. In this review, we describe the role of NGPs during cardiovascular inflammation and highlight potential therapeutic options that could be explored in the future.

## Background and rationale

Inflammation is an integral response of the human body that protects tissues challenged with sterile or nonsterile offenders [51]. The initial phase of the inflammatory response results in the production of cellular and tissue debris consisting of cellular components with protein-rich fluid. Following this initial phase of inflammation, the affected tissues must go to homeostasis, which is closely related to full functional recovery [74, 75]. However, this process does not always result in a restitution of tissue integrity, and if tissue integrity is not restored, then tissues change their appearance and function, which in itself has pathological importance. This process has been observed in several important conditions of the cardiovascular system, including atherosclerosis, inflammatory heart failure and, to some extent, myocardial ischaemia–reperfusion injury. The reperfusion phase following myocardial infarction is characterized by the infiltration of immune competent cells from the vasculature into the affected myocardial tissue [94]. Inflammation is therefore the common denominator between the development of coronary artery disease (CAD) and changes within the myocardium following reperfusion. Hence, the regulation of inflammation within the cardiovascular system potentially influences the development and outcomes of the aforementioned pathologies. Therefore, the identification of key pro- or anti-inflammatory effectors might be very important in the development of novel therapeutic strategies to reverse vascular remodelling or reduce leukocyte-mediated damage after reperfusion injury, for example.

**Fig. 1:**
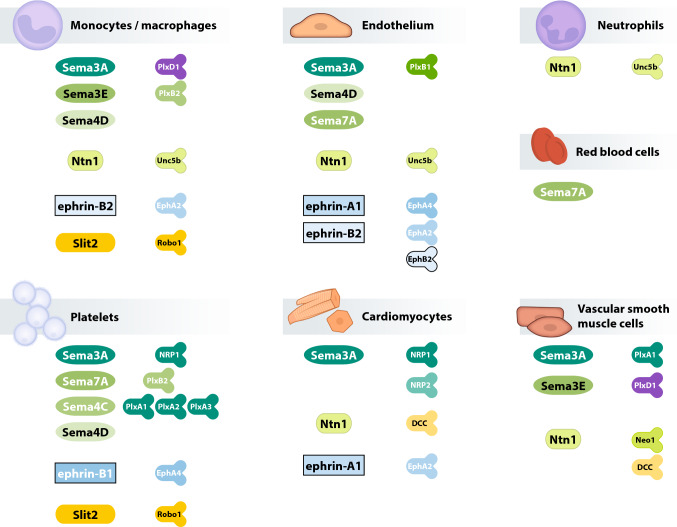
Overview of neuronal guidance protein family members with relevance in the cardiovascular system. Schematic drawing of the expression of NGP in specific tissues and sites of action (ligands in the left columns and receptors in the right columns; with *Eph* ephrin receptors, *Ntn* netrin, *Plx* plexin, *Sema* semaphorin)

According to recent studies, neuronal guidance proteins (NGPs) modulate the process of inflammation [38, 73]. This class of proteins was initially described during central and peripheral nervous system development, where NGPs serve as attractive or repulsive signals that guide axonal growth [49, 78, 95]. Subsequent studies showed that NGPs also guide the migration of leukocytes and are involved in controlling inflammatory processes [79]. NGPs exert multiple effects on the cardiovascular system, and since a large number of these proteins exist, we have included an overview of the functions of the key components of this protein class in the cardiovascular system (Fig. 1). For example, netrins are reported to modulate the tissue infiltration of immune cells, whereas semaphorins and plexins are more important during the modulation of cytokine secretion and for the control of macrophage phenotypes [53]. Inhibition or activation of NGP pathways has been shown to effectively regulate the extent of organ damage and disease outcomes [21, 54, 72]. This narrative review summarizes the current state of the literature on NGPs and the inflammatory components of coronary artery disease and consecutive myocardial ischaemia and reperfusion injury.

## The NGP family and ligand–receptor interactions

The family of NGPs is classified into major groups that are known as semaphorins, netrins, slits, ephrins and repulsive guidance molecules (RGMs). Correspondingly, NGP receptors are grouped into homologous classes. Plexins and neuropilins form a group of receptors that (co)interact with semaphorins. The netrin receptors consist of various molecules, among which the most prominent members are DCC (deleted in colorectal cancer), the DCC paralogue Neogenin-1 and the UNC5 homologues. Ephrins such as ephrin-B2 typically interact with Eph receptors, e.g., EphB2, while slits bind to Robo receptors. RGM proteins belong to the superfamily of bone morphogenetic proteins and function as ligands, receptors and co-receptors. In addition to this classical dogma of the “chaste” interaction between distinct NGP ligands and their corresponding receptors, overlaps exist. Neogenin-1 serves as a good example of one of the more promiscuous receptors interacting with both RGM-A and netrins (netrin-1 and netrin-4). Furthermore, NGPs are also ligands of receptors associated with “traditional” signal transduction, e.g., semaphorins act through integrins [62]. On the other hand, NGP receptors may essentially be activated by non-NGP ligands, e.g., VEGF-dependent coactivation of neuropilin-1 and -2 [11]. As scientific tools to investigate the interaction of proteins have become increasingly advanced, the interaction patterns of NGP ligands and receptors remain the focus of ongoing research and are likely to be further depicted in upcoming studies. Furthermore, the tissue expression patterns of NGPs show complex characteristics. In the context of CAD and acute myocardial infarction (AMI), the expression of NGPs and their corresponding receptors in the cells and tissues involved is of particular interest. These cells include cardiomyocytes (CMs), endothelial cells (ECs), vascular smooth muscle cells (VSMCs), monocytes/macrophages (MCs), fibroblasts, neutrophils (polymorphonuclear neutrophils, PMNs), red blood cells (RBCs) and platelets. Table 1 summarizes the ligand–receptor interactions of NGPs known to play a role in atherosclerosis and myocardial ischaemia.

**Table 1:** Definite interactions of neuronal guidance proteins and their corresponding receptors associated with platelet cross-talk, atherosclerosis and myocardial ischaemia

	NGP (source)	Species	Receptor	Location	Effect/signalling	Functional relevance	References
Semaphorins	Sema3A (EC, VSMC)	hu	Plexin A1, A2, A3	Platelets	Activation↓/Rac1, Cofilin	Reduced platelet activity	[32]
		mu	Plexin A1 + NRP1	VSMC	Proliferation↓, migration↓/PDGFRβ	Reduced neointimal hyperplasia	[91]
		hu	NRP1	MC	Adhesion↓, migration↓	Unknown	[81]
	Sema3E (MC, VSMC)	mu	Plexin D1	VSMC	Proliferation↓, migration↓/Rap1, PI3K, Akt	Reduced neointimal hyperplasia	[90]
	Sema4D (platelets, EC, MC)	hu	Plexin B1	EC	Adhesion↑/migration↑	Increased atherosclerotic burden [96, 100]	[47]
		hu	Plexin B2	MC	Adhesion↑/migration↑		[47]
	Sema7A (RBC, EC)	hu	β1 integrin	HSC	Megakaryocyte differentiation↓	Reduced platelet production	[30]
		hu/mu	β1 integrin	EC	VEGFR expression↑, MC adhesion↑/FAK, MEK1/2, NF-κB	Reduced intimal neovascularization, increased plaque stability	[29]
		mu	Glycoprotein Ib	Platelets	PNC formation↑	Increased infarct size	[36]
		hu	Plexin C1	EC	PMN transmigration↑	Unknown	[55]
Netrins	Netrin-1 (EC, MC)	mu	Unc5b	MC	Migration↓/Rac1	Macrophage retention within plaques	[80]
		mu	Neogenin-1	VSMC	Calcium influx↑, matrix metalloproteinase-3 activity↑	Unknown	[24]
		hu/mu	DCC	EC	VSMC migration↓, cardioprotection/eNOS induction, SIAH expression↓	Reduced neointimal hyperplasia, reduced infarct size	[42, 46, 98]
		mu	DCC	CM	Apoptosis↓, mitochondrial integrity↑/eNOS induction	Reduced infarct size, improved systolic function	[8, 9, 76]
Slits	Slit-2	hu/mu	Robo-1	Platelets	Activation↓	Reduced platelet activity	[63]
		hu/mu	Robo-1	MC	Adhesion↓, migration↓/Rho, Akt, Erk, Rac1	Reduced vascular inflammation	[56]
Ephrins	Ephrin-A1 (EC, MC)	hu	EphA2	EC	VCAM-1 expression↑/NFAT	Induction of atherogenesis	[19, 20]
		hu	EphA4	EC	MC adhesion↑, MC migration↑/Rho	Unknown	[31]
	Ephrin-B1 (EC, MC)	hu	EphA4	Platelets	Activation↓/Lyn, Fyn	Reduced platelet activity	[65]
	Ephrin-B2 (EC, MC)	hu	EphA4	EC	MC adhesion↑/Rho	Unknown	[52]
		hu/mu	EphB2	MC	Proinflammatory cytokine secretion↑	Increased vascular inflammation	[10]

NGP-mediated effects involving unknown direct ligand–receptor interactions are not listed in this table but are mentioned in the text

## Development of atherosclerosis and neuronal guidance proteins

Atherosclerosis is a complex chronic inflammatory disease that is distinguished by endothelial dysfunction, leading to the subendothelial accumulation of oxidized low-density lipoproteins (LDL), whose intracellular uptake transforms macrophages and dendritic cells into so-called foam cells [6]. VSMCs and VSMC-derived cells, e.g., macrophage-like cells, contribute to atherogenesis by promoting inflammation and modulating the extracellular matrix composition within the plaque [5]. Inflammation has been linked to the majority of pathophysiological events involved in atherosclerosis [89], and neovascularization is considered an important factor contributing to plaque destabilization [6, 82]. The effects of NGPs on endothelial biology, e.g., survival and apoptosis, are complex and have recently been reviewed by Zhang et al. [97]. Briefly, while netrins promote endothelial cell survival, semaphorins exert diverse pro-apoptotic and proliferative effects, depending on the setting and the ligand–receptor interaction.

### The semaphorins

Semaphorins contribute to endothelial dysfunction and to the earliest changes occurring in CAD. The expression of semaphorin 3A (Sema3A) in the arterial wall is repressed by pro-atherogenic stimuli such as oscillatory blood flow or inflammatory cytokines [81, 91]. Sema3A itself limits atherogenesis by inhibiting monocyte adhesion and transmigration via neuropilin-1 (NRP1), corresponding to its repulsive effect on axonal outgrowth [81]. In addition to NRP1, Sema3A binds plexin A1 and inhibits VSMC phenotype switching and neointimal hyperplasia in the damaged vasculature [91]. Interestingly, p53—a stabilizer of cellular integrity—leads to an induction of Sema3A expression, potentially reflecting a protective mechanism during states of vascular stress [91].

In contrast, semaphorin 3E (Sema3E) expression is increased in atherosclerotic plaque macrophages, and this effect is reversible when the lesions are moved to an atherosclerosis-regressive microenvironment [86]. Sema3E is accompanied by a proinflammatory macrophage expression pattern and further inhibits macrophage migration, thus trapping the cells within the lesion [86]. The effect is mediated by the plexin D1 receptor and leads to the intracellular disruption of the Rho GTPase pathway and actin cytoskeleton organization, a common mechanism regulating cell motility and migration. In a mouse model of acute carotid artery injury, however, Sema3E expression was downregulated in VSMCs, and as Sema3E acts as an inhibitor of VSMC proliferation and migration, this decrease in expression promoted neointimal hyperplasia via plexin D1 and the downstream activation of the PI3K/Akt pathway [90]. Sema3E hence serves as a good example of the diverse effects of NGPs on different cell types during similar inflammatory processes. Interestingly, serum Sema3E levels are increased in patients with atherosclerosis who are at clinical risk for adverse cardiovascular events, potentially suggesting that Sema3E represents a biomarker for vascular inflammation [66].

Semaphorin 4D (Sema4D) and its receptor plexin B1 are expressed in human endothelial cells [47, 96]. The inhibition of Sema4D leads to decreased monocyte adhesion to the endothelium, underscoring its role in the initiation of atherosclerosis [47]. In addition to its endothelial expression, Sema4D is abundantly expressed on macrophages and foam cells within the lipid-rich areas of human atherosclerotic plaques, but in its soluble form, it functions as a suppressor of oxidized LDL uptake [48]. Consequently, Sema4D knock-out mice show decreased macrophage infiltration into plaque lesions, reduced intimal neovascularization and a decreased atherosclerotic burden [96, 100]. The regulatory function of Sema4D and plexin B1 during vascular inflammation is underscored by increased expression patterns in aortic aneurysms [2].

One of the most thoroughly investigated semaphorins in the cardiovascular system is semaphorin 7A (Sema7A). Its expression is induced by oscillatory shear stress, and Sema7A facilitates increases in ICAM-1, VCAM-1 and P-selectin expression on the endothelial surface through integrin β1. These changes promote leucocyte adhesion and accelerate plaque formation [28]. Sema7A depletion in atherosclerotic mice results in a dramatically reduced accumulation of macrophages, dendritic cells and T cells within atherosclerotic plaques [29]. Consequently, Sema7A^−/−^ mice show dramatically decreased lesion sizes upon the induction of dyslipidaemia-induced atherosclerosis [28]. Furthermore, Sema7A modulates intraplaque neovascularization via β1 integrin-dependent induction of VEGFA/VEGFR expression in endothelial cells in vitro [29]. Through these mechanisms of action, Sema7A appears to fundamentally regulate vascular inflammation as a proinflammatory effector, and the attenuation of Sema7A activity might represent a protective strategy.

### The netrins

Endothelial function in the context of atherosclerosis is essentially modulated by netrin-1 (Ntn1). While the homeostatic expression of the Ntn1 receptor Unc5b appears to be a characteristic of the healthy arterial endothelium, pro-atherogenic and inflammatory stimuli suppress Unc5b expression and Ntn1 secretion [44, 61, 81]. Conversely, exogenous Ntn1 exerts direct anti-inflammatory effects on endothelial cells and attenuates the adhesion of monocytes by decreasing the production of adhesion molecules [44]. Acetylsalicylic acid (ASS), which is usually administered to patients at increased cardiovascular risk, counteracts the inflammation-induced suppression of Ntn1 secretion by the endothelium and significantly reduces plaque sizes in a murine ApoE^−/−^ model of atherosclerosis [61]. In humans, ASS counteracts the reduction in plasma Ntn1 levels after vaccination-induced endothelial dysfunction, and the effect is directly related to the extent of cyclooxygenase inhibition [40]. This finding reveals a protective effect of ASS and cyclooxygenase inhibition that extends beyond our current understanding and is driven by an NGP-mediated mechanism, preserving the endothelial barrier during vascular inflammation. During later stages of atherosclerosis, plaque-infiltrating foam cell macrophages and VSMCs secrete Ntn1 and express the Ntn1 receptor Unc5b [60, 80]. In addition to intracellular cholesterol accumulation, HIF1-α-mediated hypoxia is also an inducer of Ntn1 expression [67]. Furthermore, Ntn1 inhibits macrophage migration in vitro through Unc5b by counteracting chemoattractant-induced actin reorganization via Rac1 disruption [80]. As Ntn1 prevents macrophages from emigrating from plaques and inhibits the migration of VSMCs into lesions in mice, Ntn1 depletion in macrophages results in a dramatic decrease in the atherosclerotic burden [80, 93]. Notably, while Ntn1 induces remodelling of the extracellular vascular matrix by VSMCs, early Ntn1 administration reduces neointimal hyperplasia, vascular inflammation and the atherosclerotic lesion size [24, 34]. Both the neogenin-1 and the DCC receptor are responsible for signal transduction in VSMCs, and the latter induces endothelial nitric oxide synthase (eNOS) expression [24, 46]. Based on these results, Ntn1 functions as a protective anti-inflammatory ligand during early atherogenesis but might impair vascular regeneration during later stages of lesion development. This is consistent with clinical findings of increased plasma Ntn1 levels in patients at risk of endothelial dysfunction compared to decreased Ntn1 concentrations in patients with progressed forms of atherosclerotic disease [35, 57]. Hence, Ntn1 and its receptors must be considered molecular targets for anti-atherosclerotic therapies.

### The ephrins

A wide variety of ephrins and ephrin receptors are expressed in human endothelial cells, monocytes and atheroma foam cells [31, 70, 71]. However, only a few of these proteins play substantial roles in atherosclerosis. The induction of ephrin-A1 expression by pro-atherogenic stimuli such as oxidized LDL leads to increased monocyte adhesion after it interacts with endothelial EphA4 and EphA2 receptors [20, 31]. EphA2 activation induces VCAM-1 expression via NFAT signalling [19]. Although plaque macrophages also express EphA2, only endothelial EphA2 is responsible for macrophage invasion and subsequent atherosclerotic lesion growth, and its suppression appears to be a characteristic of a healthy and uninflamed vascular barrier [17]. Endothelial ephrin-B2 also attracts monocytes and activates EphB2 receptors upon their adhesion [10]. Subsequently, monocyte attractant chemokines such as CCL2 and IL-8 are secreted and participate in a potent mechanism involved in the initiation of atherosclerosis. Monocyte adhesion is further facilitated by the interaction of inflammatory ephrin-B2-producing macrophages with the endothelial EphA4 receptor [52, 64]. Taken together, these findings stress the complex feedback mechanisms involving ephrin-B2 between the endothelium and monocytes during atherogenesis. As part of an interventional approach, chondroitin sulphate was able to counteract the TNFα-mediated endothelial induction of ephrin-B2 expression in mice, consequently leading to decreased macrophage/foam cell invasion and smaller atherosclerotic lesions [52]. Through similar mechanisms of action, ephrins contribute to atherogenesis—mainly by promoting the migration of innate immune cells—and the inhibition of ephrin signalling might represent a measure to antagonize the (micro)-inflammatory changes leading to plaque progression.

### Slit-2 and Robo-1

Slit-2 represents the only non-semaphorin, non-netrin and non-ephrin NGP with a reported role in atherogenesis. Similar to its effect on platelet function, Slit-2 suppresses monocyte migration and adhesion to vascular endothelial cells in atherosclerotic lesions, presumably through the Robo-1 receptor [56]. Interestingly, monocytes from patients with CAD showed reduced levels of the Robo-1 transcript and were subsequently less responsive to Slit-2 inhibition of cell migration [56]. In rats with acute arterial injury, Slit-2 also repelled monocyte infiltration into the lesioned vessel wall [45]. Slit-2 is also known to inhibit leukocyte chemotaxis induced by chemotactic factors. This inhibition of chemokine-induced chemotaxis was reversed when cells were reconstituted with soluble Robo-1 [92].

**Fig. 2:**
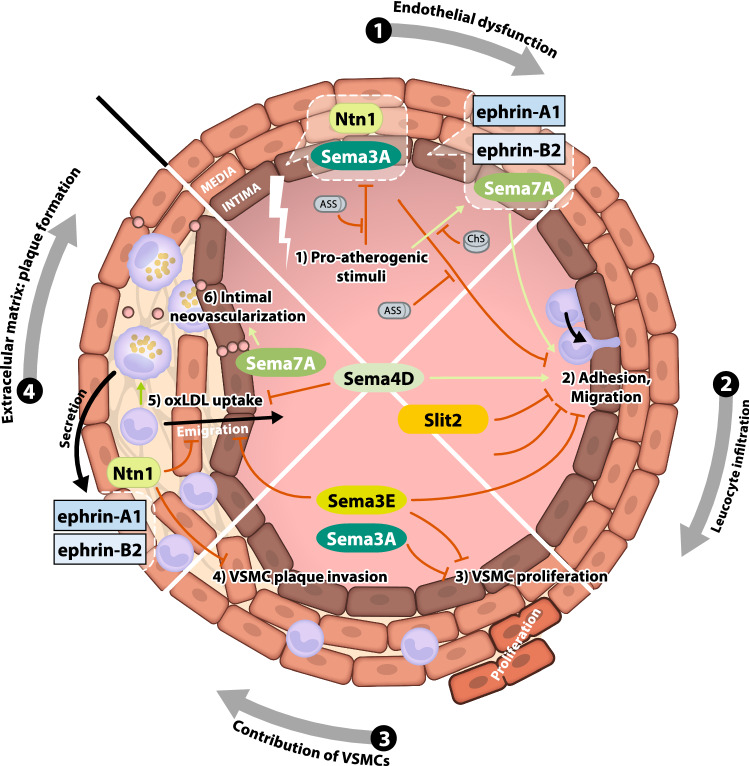
Role of NGPs in the development of atherosclerosis. Sema3A expression is decreased in the stressed endothelium, Sema3A overexpression successfully inhibits VSMC proliferation and migration; Sema3A also inhibits monocyte adhesion and migration. Sema4D and its receptor PlxB1 promote monocyte adhesion to the endothelium, impair platelet aggregation and regulate the atherosclerotic lesion size. Sema7A regulates the expression of ICAM-1, VCAM-1 and P-selectin on the endothelial surface, promoting leucocyte adhesion and plaque formation. Sema7A also modulates intraplaque neovascularization via β1 integrin-dependent induction of VEGFA/VEGFR expression in endothelial cells. Ntn1 regulates the adhesion molecules VCAM-1, ICAM-1 and E-selectin and prevents macrophages from emigrating from plaques. Ntn1 further inhibits the migration of VSMCs into lesions. Ephrin-A1, Ephrin-B1 and Ephrin-B2 regulate the migration of monocytes into the vascular wall (with *Ntn* netrin, *Plx* plexin, *Sema* semaphorin)

Figure 2 summarizes the regulatory functions of NGPs during the development and progression of atherosclerotic lesions. Unsurprisingly, a vast majority of the effects comprise the repulsion or attraction of immune cells and VSMCs, similar to their effects on neuronal guidance during embryogenesis.

## Myocardial ischaemia–reperfusion injury and neuronal guidance proteins

IR injury is defined as reduced target organ perfusion leading to ischaemia, which is followed by the restoration of blood flow called reperfusion. While acute ischaemia is responsible for the initial stage of metabolic and inflammatory changes within the affected area, the infiltration of innate immune cells—mainly neutrophils—into the infarct zone during reperfusion aggravates sterile inflammation and is responsible for the majority of collateral tissue injury [94]. As this phenomenon may describe the mechanism driving organ damage after IR, the immunomodulatory effects of NGPs and their receptors are a promising target to alter the extent of IR injury and patient outcomes. Numerous reports have investigated the effects of NGPs on myocardial infarction, with a focus on the participating cell types.

### The semaphorins

The semaphorins attenuate and aggravate tissue injury after myocardial infarction. Sema3A belongs to the cardioprotective subgroup, and its administration leads to a decreased infarct size, sustained left ventricular systolic function, a reduction in post-ischaemic electrical remodelling and the occurrence of ventricular arrhythmias [26, 68, 87]. Again, dampening the inflammatory response after AMI appears to be the key mechanism. Sema3A inhibits monocyte recruitment into the infarct zone and promotes their conversion into resolution-phase macrophages [68]. Monocytes obtained from patients during myocardial wound healing show increased Sema3A expression compared to cells obtained at the onset of AMI [68]. Sema3A is also associated with increased myocardial apoptosis, potentially limiting DAMP-driven inflammation of the surrounding tissue [25].

In contrast, Sema4D and Sema7A are proinflammatory semaphorins functioning during myocardial ischaemia. Plasma Sema4D levels are elevated in patients with acute coronary symptoms and correlate with C-reactive protein levels, potentially indicating a biomarker function [23]. Sema7A is upregulated in the endothelium during hypoxia and facilitates the transmigration of PMNs via plexin C1 [55]. Furthermore, Sema7A is shed from the surface of RBCs during myocardial IR and activates the platelet GPIb receptor, resulting in the formation of platelet–neutrophil complexes [36]. These complexes increase the degree of tissue injury, and the inhibition of Sema7A alleviates inflammatory injury to the heart following IR [36].

The role of neuropilin receptors requires further investigation, as they interact promiscuously with semaphorins and VEGF. NRP1 was shown to preserve endothelial barrier function, but undergoes degradation during hypoxia [3]. Compared to Sema3A, NRP1 inhibits adverse electrical remodelling after AMI in rats, which show reduced infarct sizes and are less prone to ventricular tachycardia [88]. Furthermore, NRP2 functions as a VEGFB receptor within the heart, promoting angiogenesis, anti-apoptotic and cardioprotective metabolic shifts in the ischaemic myocardium [39].

### The netrins

Similar to vascular inflammation, the vast majority of reports on the roles of netrins in myocardial ischaemia have investigated netrin-1, which is downregulated following myocardial ischaemia [12, 50]. Nitric oxide (NO)—a key molecule preserving endothelial function—was identified as a major mediator of the cardioprotective effects of Ntn1. Ntn1 induces NO production through the DCC receptor followed by Erk and eNOS activation, and Ntn1 administration leads to a substantial NO-dependent reduction in myocardial infarct sizes ex vivo [42, 98]. The concept of post-infarct tissue conservation by Ntn1 was further shown in diabetic mice [33]. As the administration of small synthesized peptides containing Ntn1 domains successfully induced NO production in an animal model, pharmacological approaches have been developed to mimic the cardioprotective effects of Ntn1 [42]. The Ntn1–DCC–NO axis further attenuates mitochondrial superoxide production and preserves mitochondrial integrity in cardiomyocytes post-conditioned with Ntn1 after IR injury [8, 9, 76]. Subsequent studies identified SIAH—a ubiquitin ligase—as a mediator of Ntn1-induced cardioprotection in endothelial cells [43]; NO production induced by Ntn1 downregulated SIAH expression and therefore decreased proteasomal degradation of the DCC receptor, further increasing protective NO synthesis in a feed-forward manner. Ntn1 administration preserves cardiac function and decreases the extent of tissue damage in a mouse model of ischaemia and consecutive heart transplantation [50]. These changes are accompanied by a PPARγ-dependent reduction in cardiomyocyte apoptosis and leukocyte infiltration while shifting the phenotype of infiltrating macrophages towards the pro-resolutionary phenotype [50]. Combined with the observed protective effect of Ntn1 on oxidative stress [14], Ntn1 appears to support post-inflammatory resolution and therefore plays a pivotal role in tissue regeneration. Correspondingly, post-AMI physical exercise treatment in rats induced Ntn1 expression, which was associated with decreased myocardial fibrosis and improved cardiac function [12].

When examining the roles of Ntn1 signal transduction other than in inducing NO production, Ntn1 signalling during myocardial ischaemia appears to be mainly dependent on the Unc5b receptor. The in vitro inhibition of Unc5b dramatically reduces neutrophil transmigration [37]. This decrease translated into the Unc5b-dependent inhibition of neutrophil extravasation associated with decreased infarct sizes after myocardial IR in vivo. The effects were abolished by neutrophil depletion prior to coronary artery occlusion [37]. These results underscore the significance of the effects of the Ntn1–Unc5b axis on PMN-mediated myocardial IR injury.

### The ephrins

Ephrins and ephrin receptors affect myocardial ischaemia–reperfusion injury, but the mechanisms and resulting effects are more heterogeneous and strongly depend on the observed tissue and time point. Ephrin-A1 expression in the myocardium is decreased following myocardial infarction in mice, and exogenous ephrin-A1 dramatically diminishes tissue injury [13, 41]. Both the degree of myocardial apoptosis, which is presumably triggered via increased Akt phosphorylation, and neutrophil infiltration are reduced following ephrin-A1 treatment [13]. Furthermore, ephrin-A1 administration leads to significant functional improvements, as shown by echocardiographic measurements, in a murine model of prolonged myocardial IR, supporting its overall cardioprotective effect [16]. Depletion of EphA2, a receptor for ephrin-A1, results in increased infarct sizes and tissue inflammation following permanent coronary occlusion [59]. EphA2 knock-out mice present with an echocardiographic phenotype of decreased cardiac function at baseline, which further deteriorates after AMI compared to controls, suggesting an important role for the ephrin-A1–EphA2 axis in healthy myocardial homeostasis [59]. Interestingly, although the depletion of EphA2 leads to significantly increased mortality following myocardial infarction, it abolishes the effect of hyperglycaemia on the extent of tissue injury, as observed in WT animals [15]. The loss of the EphA2 receptor results in inadequate post-infarct leucocyte infiltration and wound healing, superimposing the adverse effects of diabetic metabolism on the heart. Ephrin-B2 is another ephrin that influences post-ischaemic cardiac remodelling and fibrosis. The shRNA-mediated inhibition of ephrin-B2 during AMI counteracts its increased expression in fibroblasts and reduces interstitial myocardial fibrosis while preserving systolic function [77]. Here, ephrin-B2 drives cardiac fibroblasts towards myofibroblast differentiation via Jak2/Stat3 and TGF-β/Smad3 signalling, which was shown to be major pathways involved in cardiac fibrosis [77].

**Fig. 3:**
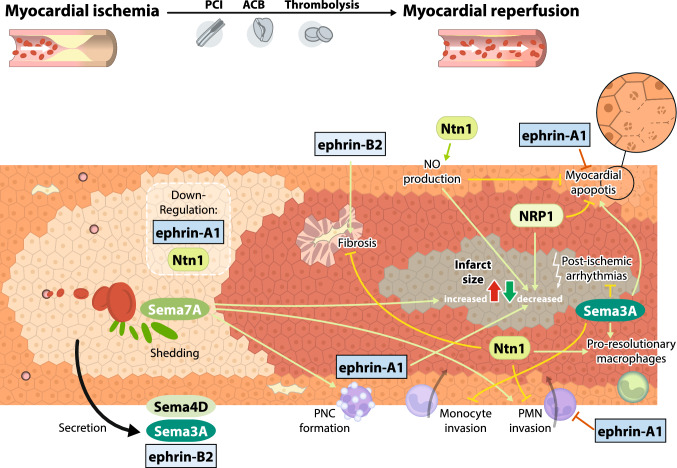
Role of NGPs in inflammation during myocardial ischaemia–reperfusion injury. Sema3A expression is induced by AMI, dampens the inflammatory response and decreases infarct size. Sema4D levels are elevated in patients presenting with acute coronary syndrome, reflecting the inflammatory component of myocardial ischaemia. Sema7A activates the platelet GPIb receptor during IR injury, resulting in the formation of platelet–neutrophil complexes and the aggravation of tissue injury. Neuropilin-1 improves electrical remodelling at the infarct border, reduces infarct sizes and functions as a VEGFB receptor, promoting angiogenesis, anti-apoptotic and cardioprotective metabolic shifts in the ischaemic myocardium. Ntn1 substantially reduces the infarct size through NO and the recruitment of pro-resolutionary macrophages. Ephrin-A1 expression is decreased following myocardial infarction in mice, and exogenous ephrin-A1 dramatically diminishes tissue injury and leads to functional improvement. (with *Ntn* netrin, *NO* nitric oxide, *Plx* plexin, *Sema* semaphorin)

An overview of the functional effects of NGPs on myocardial ischaemia and reperfusion injury is displayed in Fig. 3.

### MicroRNA-targeted efforts

As genetic-based medical therapy has been a topic of debate over the last few decades, multiple RNA-based treatment approaches modulating NGP expression during myocardial ischaemia have been tested in the experimental setting and provided further insights into the underlying mechanisms of NGPs. For instance, to date, the role of ephrin-A3 has only been investigated by downregulation via miRNA-210, which leads to improved cardiac function in a murine model of myocardial infarction [27]. Interestingly, cardiac progenitor cells show enrichment of miRNA-210 in their extracellular vesicles (EVs), and EV secretion during murine AMI correspondingly results in cardioprotective effects and induces post-ischaemic angiogenesis [4, 83]. The secretion of EVs represents a fairly novel concept in the contribution to the post-ischaemic response. As human and mouse endothelial cells are known to secrete EVs in response to AMI, their miRNA content influences NGP expression by predominantly downregulating plexin B2, particularly in monocytes [1]. Together with an induction of integrin expression, these endothelial EVs promote increased monocyte mobilization from the spleen, potentially facilitating the innate immune response within the ischaemic myocardium [1]. Finally, the miRNA-34 family is upregulated by myocardial ischaemia in mice and humans and links Sema4B—one of its targets—to improved functional outcomes [7].

## Neuronal guidance proteins and thromboinflammation

Thromboinflammation comprises the involvement of platelets in inflammatory processes, such as the promotion of neutrophil extravasation or the induction of cytokine secretion, in addition to or beyond their function in primary haemostasis [69]. This process is essential for various states of cardiovascular inflammation, including sepsis, acute respiratory distress syndrome and the development of thrombosis [58]. Hence, the known interactions between NGPs and platelets might be of scientific and clinical interest to ameliorate these conditions.

NGP affects platelet physiology even during the early stages of development, which is known as thrombopoiesis. Sema7A represses megakaryocyte differentiation via integrin β1 and simultaneously induces proinflammatory cytokine secretion in haematopoietic progenitor cells [30]. Conceivably, during states of vascular inflammation, haematopoietic stem cell differentiation is thereby shifted from the generation of platelets towards the production of classic immune cells. In vitro miRNA-based inhibition of plexin B2 in platelet-like cells leads to increased thrombotic platelet reactivity, implying that the receptor and possibly its ligand semaphorin 4C (Sema4C) function as regulators of platelet activity [22]. Similar results were observed for Sema3A acting through its receptors plexin A1, A2 and A3, inhibiting both aggregation and granule secretion [32].

**Fig. 4:**
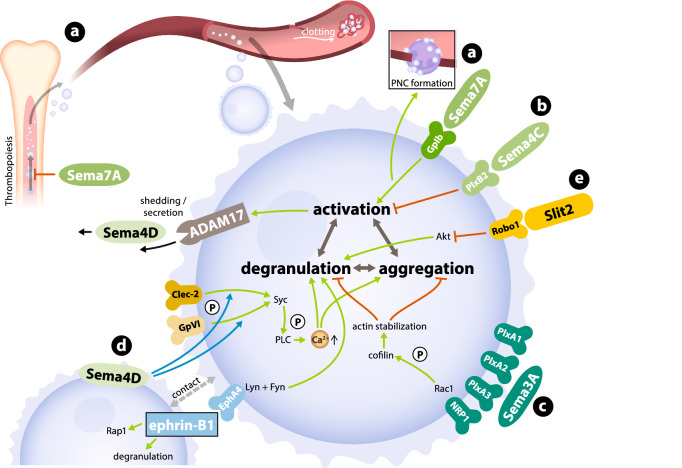
Interaction between platelets and NGPs. **a** Sema7A influences megakaryopoiesis and reduces differentiation and megakaryocyte and platelet production. It also interacts with the platelet GPIb receptor and activates platelets through this receptor. **b** PlxB2 functions as a receptor for Sema4C and regulates the activation and thrombotic activity of platelets. **c** Sema3A interacts with PlxA1-3 and inhibits the secretion of granules and aggregation of platelets. **d** Sema4D phosphorylates glycoprotein VI and Syk as downstream targets of Clec-2. **e** Slit-2 was shown to inhibit platelet adhesion, aggregation and granule secretion via the Robo-1 receptor (with *Sema* Semaphorin, *Plx* Plexin)

Despite these inhibitory effects, some semaphorins may also be essential for platelet activation and thrombus formation. Sema4D contributes to collagen-induced platelet activation and the subsequent increase in intracellular Ca^2+^ concentrations in mice [85]. A defect in the Sema4D-dependent phosphorylation of glycoprotein VI and the Clec-2 downstream target Syk and the resulting lack of activated PLC are responsible for this effect [84]. Sema4D also contributes to thrombus formation, which requires cell-to-cell contacts between platelets, possibly through a synapse-like mechanism involving membrane-bound Sema4D [84, 85]. Similar results were obtained for ephrin-B1 and its receptor EphA4 during platelet adhesion [65]. Astonishingly, platelets from Sema4D knock-out mice that were activated by dyslipidaemia show markedly decreased aggregation compared to wild-type platelets [100]. Sema4D is both expressed on the surface of activated platelets and secreted in its soluble form, which potentially exerts anti-inflammatory effects on the surrounding vasculature [18, 99]. Slit-2—a suppressor of immune cell migration—was shown to inhibit platelet adhesion, aggregation and granule secretion via the Robo-1 receptor [63]. Collectively, these findings underscore the cross-talk between platelets and innate immune cells mediated by NGPs in the context of vascular inflammation and suggest that NGPs represent a therapeutic target before or during arterial occlusion. Figure 4 summarizes these effects at the cellular and molecular levels. As literature on this topic is still sparse, further insights are needed to understand its complete translational potential.

## Summary and perspectives

The essential role of neuronal guidance proteins in modulating inflammation during the course of coronary artery disease and myocardial ischaemia has been documented by a number of reports. Leucocyte–endothelial interactions, the migration of innate immune cells and extracellular tissue composition appear to be critical factors contributing to this process. In addition, the interaction of NGPs with platelets during thromboinflammation might also reflect a major area of NGP action during cardiovascular inflammation. Netrin-1 and semaphorin 7A are meaningful examples of the spectrum of these effects. The translation of these findings into patient care might have clinical importance in the future. Extended translational concepts employing techniques such as miRNA, siRNA or adeno-associated virus-based administration should focus on modulating these NGP pathways. Therefore, these pathways should be further deciphered since they have been incompletely characterized to date. In summary, advancing experimental research and elucidating the underlying mechanisms involving all NGP family members in the context of inflammatory changes that result in heart disease will prompt the development of new therapeutic strategies in the future.

## Abbreviations

AMI: Acute myocardial infarction
ApoE: Apolipoprotein E
ASS: Acetylsalicylic acid
Ca^2^^+^: Ionized calcium
CAD: Coronary artery disease
CCL2: CC-chemokine ligand 2
Clec-2: C-type-lectin-like-2
CM: Cardiomyocytes
CREB: CAMP response element-binding protein
CRP: C-reactive protein
DAMP: Damage-associated molecular pattern
DCC: Deleted in colorectal cancer
EC: Endothelial cell(s)
eNOS: Endothelial nitric oxide synthase
EphA2: Ephrin type-A receptor 2
EphA4: Ephrin type-A receptor 4
Erk: Extracellular signal-regulated kinase
EV: Extracellular vesicle(s)
FAK: Focal adhesion kinase
GTP: Nucleotide guanosine triphosphate hydrolase
HIF-1α: Hypoxia-inducible factor 1α
Hu: Human
ICAM-1: Intercellular adhesion molecule 1
IL-8: Interleukin 8
IR: Ischaemia and reperfusion
Jak2: Janus kinase 2
LDL: Low-density lipoprotein(s)
LPS: Lipopolysaccharides
MC: Monocytes/macrophages
MEK1/2: Mitogen-activated protein kinase 1/2
miRNA: Microribonucleic acid
MSC: Mesenchymal stem cell
Mu: Murine
NFAT: Nuclear factor of activated T cells
NF-κB: Nuclear factor kappa-light-chain-enhancer of activated B cells
NGP(s): Neuronal guidance protein(s)
NO: Nitric oxide
NRP1: Neuropilin-1
Ntn1: Netrin-1
PMN: Polymorphonuclear neutrophil
PNC(s): Platelet–neutrophil complex(es)
PPARγ: Peroxisome proliferator-activated receptor γ
Rap1: Ras-related protein 1
RBC: Red blood cells
RGM: Repulsive guidance molecule
Sema3A: Semaphorin 3A
Sema3E: Semaphorin 3E
Sema4C: Semaphorin 4C
Sema4D: Semaphorin 4D
Sema7: Semaphorin 7A
shRNA: Small hairpin ribonucleic acid
siRNA: Small interfering ribonucleic acid
Smad3: Mothers against decapentaplegic homologue 3
Stat3: Signal transducers and activator of transcription 3
Syk: Spleen tyrosine kinase
TGF-β: Transforming growth factor β
TNFα: Tumour necrosis factor α
VCAM-1: Vascular cell adhesion molecule 1
VEGF (A/B): Vascular endothelial growth factor (A/B)
VEGFR: Vascular endothelial growth factor receptor
VSMC: Vascular smooth muscle cell
